# What Is the Role of Thalamostriatal Circuits in Learning Vocal Sequences?

**DOI:** 10.3389/fncir.2021.724858

**Published:** 2021-09-22

**Authors:** Lei Xiao, Todd F. Roberts

**Affiliations:** Department of Neuroscience, UT Southwestern Medical Center, Dallas, TX, United States

**Keywords:** songbird, reinforcement learning, vocal learning, cholinergic interneurons, striatum

## Abstract

Basal ganglia (BG) circuits integrate sensory and motor-related information from the cortex, thalamus, and midbrain to guide learning and production of motor sequences. Birdsong, like speech, is comprised of precisely sequenced vocal elements. Learning song sequences during development relies on Area X, a vocalization related region in the medial striatum of the songbird BG. Area X receives inputs from cortical-like pallial song circuits and midbrain dopaminergic circuits and sends projections to the thalamus. It has recently been shown that thalamic circuits also send substantial projections back to Area X. Here, we outline a gated-reinforcement learning model for how Area X may use signals conveyed by thalamostriatal inputs to direct song learning. Integrating conceptual advances from recent mammalian and songbird literature, we hypothesize that thalamostriatal pathways convey signals linked to song syllable onsets and offsets and influence striatal circuit plasticity *via* regulation of cholinergic interneurons (ChIs). We suggest that syllable sequence associated vocal-motor information from the thalamus drive precisely timed pauses in ChIs activity in Area X. When integrated with concurrent corticostriatal and dopaminergic input, this circuit helps regulate plasticity on medium spiny neurons (MSNs) and the learning of syllable sequences. We discuss new approaches that can be applied to test core ideas of this model and how associated insights may provide a framework for understanding the function of BG circuits in learning motor sequences.

## Introduction

**Figure 1:**
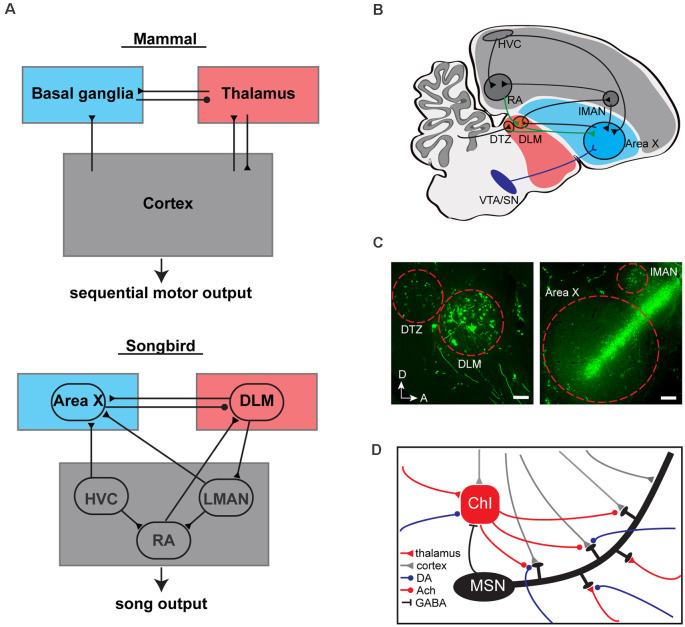
Basal ganglia (BG)-thalamocortical loops in mammals and songbirds. **(A)** BG-thalamocortical loops are evolutionarily conserved in songbirds and mammals to drive sequential motor (or song) output. Circles and triangles represent GABAergic and glutamatergic inputs, respectively. **(B)** Schematic of the BG-thalamocortical loops in songbirds. Area X receives multiple inputs from song circuits: glutamatergic inputs from cortical like song circuits HVC and LMAN; glutamatergic inputs from thalamic circuits DLM and DTZ; dopaminergic inputs from midbrain circuits VTA and SN. Circles, triangles, and Y-shaped projections represent GABAergic, glutamatergic, and dopaminergic inputs, respectively. **(C)** Representative sagittal sections through the thalamus (left panel) and Area X (right panel) when fluorescent tracers were injected into Area X (dextran, Alexa Fluor 488) of adult zebra finch. Left panel, parasagittal section through retrogradely labeled thalamic circuits DLM and DTZ, scale bar, 100 μm. Right panel, parasagittal section through retrogradely labeled cortical circuit LMAN, scale bar, 200 μm. D, dorsal; R, rostral. **(D)** Schematic of striatal microcircuits in mammals. The main glutamatergic inputs to the striatum are from the cortex (gray triangles) and thalamus (red triangles). Both inputs target overlapping populations of MSNs as well as ChIs and other interneurons (not shown). Modulatory inputs from dopaminergic (DA, blue circles) and cholinergic inputs from ChIs (Ach, red circles) are involved in modulating the synapses onto MSNs. MSNs provide GABAergic inputs to ChIs (black line) and other interneurons (not shown). ChI, cholinergic interneuron. MSNs, medium spiny neurons.

The ability to adeptly sequence motor actions is central to animal survival, coordinated movement, and communication. Basal ganglia-thalamocortical loops have been demonstrated to be essential for the learning, coordination, and execution of sequenced motor actions (Jin et al., 2014; Tecuapetla et al., 2016; Park et al., 2020). The precise movements and sequencing of actions involved in producing learned vocalizations are one of the clearest and most readily addressable natural behaviors that can be used to examine how motor sequences are learned and controlled by the brain (Brainard and Doupe, 2002; Fee et al., 2004). Two major glutamatergic inputs to the striatum, the largest principal component of the basal ganglia, have been proposed to drive striatal activity that regulates vocal sequences: the corticostriatal and the thalamostriatal inputs (Kemp et al., 1971; Gerfen and Wilson, 1996; Smith et al., 2004; Figure 1A).

In contrast to the well-recognized functions of corticostriatal input in sequential behaviors (Hikosaka et al., 1999; Rothwell et al., 2015; Kupferschmidt et al., 2017), it is less clear whether and how the thalamostriatal input is involved in the motor learning and performance, especially when the sequences of motor output are fast and complex (Parker et al., 2016; Díaz-Hernández et al., 2018). An increasing body of literature suggests that cortical circuits are necessary for initial learning of motor sequence and, once learned, subcortical circuits may be sufficient for their expression (Kawai et al., 2015; Kupferschmidt et al., 2017; Wolff et al., 2019). Yet, how signals conveyed by distinct cortical and/or subcortical inputs to the striatum are integrated during learning and potentially decoupled once expert performance is achieved is still unclear. For example, how is time-sensitive information incorporated in the striatum from cortical and subcortical inputs? How do these pathways regulate synaptic plasticity during learning and once motor sequences are learned? Do thalamostriatal and corticostriatal circuits convey redundant, complementary, or distinct information to the striatum?

Many experimental paradigms have been developed in rodents to tackle these and related questions (Kawai et al., 2015; Rueda-Orozco and Robbe, 2015; Díaz-Hernández et al., 2018; Hidalgo-Balbuena et al., 2019). However, a coherent hypothesis with testable predictions for the role of thalamostriatal circuits in learning and performance of sequential behaviors has been slow to develop. The recent identification of thalamostriatal circuits in the songbird presents an opportunity to use song learning to generate hypotheses and predictions for thalamostriatal circuit function (Nicholson et al., 2018; Pidoux et al., 2018). Songbirds learn a sequence of vocal elements (song) from a vocal model (tutor) as juveniles and use this song in adulthood to attract mates and defend territory (Zann, 1996; Searcy and Beecher, 2009; Bradbury and Vehrencamp, 2011; Ikeda et al., 2020). The song is learned naturally during development. Therefore, the learning process is free from external reinforcers typically used in laboratory settings, such as food or water, which are known to impact dopaminergic reward circuits (Arbuthnott and Wickens, 2007; Moss and Bolam, 2008; Rice and Cragg, 2008). In addition, the song is highly quantifiable and trackable. Male zebra finches, for example, produce thousands of highly stereotyped song renditions daily which, when combined with neural recordings and/or circuit manipulations, permit detailed study of how brain circuits control behavioral learning and performance. Lastly, the neural circuits underlying song learning and production are well characterized and cellular homologies between song circuits and mammalian circuits are starting to be revealed (Farries, 2004; Pfenning et al., 2014; Gadagkar et al., 2016; Vallentin et al., 2016; Hisey et al., 2018; Xiao et al., 2018; Colquitt et al., 2021; Xiao et al., 2021).

From this perspective, we discuss the potential roles of the thalamostriatal pathway in vocal learning, propose a testable hypothesis invoking recent breakthroughs in rodents and songbirds, and discuss new methodologies that can be applied to test core ideas in our model. Rather than a detailed review of the state of the field, we attempt to provide a new avenue for understanding the function of BG circuitry in directing sequential behaviors.

### Role of Inputs to Area X in Vocal Learning and Production

Area X is a specialized song nucleus within the striatum that receives inputs from cortical-like pallial song circuits [HVC (used as a proper name, formerly known as high vocal center) and LMAN (lateral part of the magnocellular nucleus of anterior neostriatum)] and dopaminergic input from midbrain circuits [VTA (ventral tegmental area) and SN (substantia nigra)]. Area X sends projections to the thalamic nucleus DLM (dorsolateral nucleus of the anterior thalamus) *via* pallidal-like cells and DLM, in turn, sends projections to the pallial song nucleus LMAN. Area X, DLM, and LMAN constitute the anterior forebrain pathway (AFP), a basal ganglia-thalamocortical loop associated with learning and sequencing of birdsong (Figure 1B). Both cortical input from HVC and midbrain input from VTA/SN in Area X are needed for song learning in juvenile birds but not needed for continued production of learned song in adulthood (Scharff et al., 2000; Miller et al., 2015; Hisey et al., 2018; Sánchez-Valpuesta et al., 2019). Ablation of either input in juvenile birds causes deficits in the imitation of tutor song, resulting in a less stereotyped acoustic features and sequencing of syllables. In contrast, individual lesions of either HVC neurons projecting to Area X or dopaminergic inputs in adult birds have little impact on the overall structure of the learned song. Nonetheless, lesions of dopaminergic inputs to Area X have been shown to disrupt the ability to modify song acoustic features in response to disruptive auditory feedback (Hoffmann et al., 2016; Saravanan et al., 2019). Overall, these observations align with findings in rodents indicating that corticostriatal pathways are necessary for initial learning but can be dispensable for performance once motor sequences are well learned (Tecuapetla et al., 2016; Kupferschmidt et al., 2017).

In contrast to a diminished role of the corticostriatal pathway in the performance of learned motor sequences, thalamostriatal inputs may be necessary for both learning and subsequent expert performance of motor sequences. Ablation of thalamic inputs to the dorsolateral striatum (DLS) prevents naïve rats from learning a new motor sequence and revert the performance of motor sequences in expert rats to levels similar to those observed in the early phases of learning (Hidalgo-Balbuena et al., 2019; Wolff et al., 2019). These results argue that thalamostriatal projections may be relevant in driving the dorsal striatum and necessary to perform a sequence of learned motor actions.

Are there thalamic projections to Area X? Tracer injections in Area X retrogradely label neurons in DLM and adjacent thalamic nuclei (Bottjer et al., 1989; Castelino et al., 2007; Person et al., 2008; Gale and Perkel, 2010; Pidoux et al., 2018; Figure 1C). However, retrograde labeling in the thalamus could result from tracer efflux into pallial regions immediately dorsal to Area X, including LMAN, which are known to receive inputs from the thalamus. Thus, the validity of thalamicalamic projections to Area X has until recently been controversial. Viral vector labeling of presynaptic axon terminals from DLM and DTZ (dorsal thalamic zone) has now been used to confirm that DLM and DTZ do indeed provide direct projections to Area X (Nicholson et al., 2018).

These two thalamostriatal projections, from DLM and DTZ, likely carry different types of information to Area X. In addition to reciprocal projections with Area X, DLM receives excitatory input from the motor cortical-like song circuit RA (robust nucleus of the arcopallium), which transmits premotor signals necessary for song learning (Wild, 1993; Vates et al., 1997; Luo and Perkel, 2002; Goldberg et al., 2012; Goldberg and Fee, 2012). DTZ, on the other hand, receives input from deep cerebellar nuclei (Person et al., 2008; Pidoux et al., 2018). Although much remains to be learned about the function of these two thalamostriatal pathways in song production and song learning, current evidence indicates that they may each play important roles in song learning and song motor control. Lesions of DLM in adult birds disrupt AFP-driven song initiation and lesions in juvenile birds disrupt early stages of vocal production (Goldberg and Fee, 2011; Chen et al., 2014). Much less is currently known about DTZ, but lesions of deep cerebellar nuclei have also been shown to disrupt song imitation in young birds (Pidoux et al., 2018). For the remainder of this perspective, we will focus on the potential roles of the DLM-Area X circuit in vocal learning.

### What Signal is Encoded by Thalamostriatal Pathways in Songbirds?

Song syllables are the essential behavioral units of a song. For example, song truncation following a startling stimulus tends to occur at the end of individual syllables and not mid-syllable, suggesting syllable-level chunking of motor programs (Cynx, 1990). Songbirds learn to arrange syllables into sequences independently from learning the spectral content of each individual syllable, supporting the idea that syllables represent a meaningful behavioral unit (Tchernichovski et al., 2001; Liu et al., 2004; Ravbar et al., 2012; Lipkind et al., 2013, 2017). Moreover, Area X appears to be an important contributor to learning and controlling song syllable syntax. Lesions of Area X in juvenile birds result in birds with variable song syntax as adults (Sohrabji et al., 1990; Scharff and Nottebohm, 1991; Goldberg and Fee, 2011). Knockdown of the speech-linked gene FoxP2, overexpression of the mutant gene fragment that causes Huntington’s disease, and optogenetic excitation of dopaminergic inputs in Area X all cause progressive disruptions in song syllable sequencing, including repetition of song syllables and disruptions in song syntax in adult birds (Tanaka et al., 2016; Xiao et al., 2021).

Nonetheless, reinforcement-based models for song learning largely focus on learning features of song syllables rather than syllable sequences (Fee and Goldberg, 2011; Fee, 2012; Chen and Goldberg, 2020; Kornfeld et al., 2020). Therefore, from a circuit perspective, it is not clear how chunked motor programs associated with syllable level representations might be acquired during development. It has been proposed that temporal information associated with sequential events could emerge from the interaction of cortical and thalamic inputs to the BG (Mello et al., 2015; Paton and Lau, 2015). Yet, the neural signals used to encode information at different time scales are not known. *In vitro* electrophysiological recordings in mammals suggest that corticostriatal and thalamostriatal pathways encode information in temporally distinct ways (e.g., low vs. high release probability; synapses were facilitated vs. depressed by repetitive stimulation) and therefore constrain how they inform striatal circuits (Ding et al., 2008). More recently, *in vivo* work characterizing the sound-evoked responses of thalamostriatal and corticostriatal neurons further suggests that these pathways can convey different yet complementary auditory information to the striatum (Ponvert and Jaramillo, 2019). Although both pathways encode sound frequency information, corticostriatal inputs provide a more accurate representation of amplitude modulation rate and thalamostriatal inputs convey information about the precise timing of acoustic events. We propose that Area X receives three distinct streams of information about a song: a detailed timestamp for each moment in the song from HVC, a signal about the variability of spectral content at each moment from LMAN, and information about syllable onsets and offsets that permits syllable-level chunking of behavior from DLM. Together, these three inputs may provide essential substrates to support the learning of spectral features in syllables as well as syllable sequences.

During singing, Area X neurons appear to encode information associated with the timing of specific moments in the song. Individual medium spiny neurons (MSNs) in Area X exhibit sparse activity (1–4 bursts/motif) that is precisely time-locked to particular points in song (Farries and Perkel, 2002; Goldberg et al., 2010; Goldberg and Fee, 2011; Woolley et al., 2014). The distribution of activity across the population of MSNs is thought to cover the entire sequence of song syllables in a bird’s song motif (Farries and Perkel, 2002; Goldberg et al., 2010; Fee and Goldberg, 2011; Woolley et al., 2014). This activity pattern may reflect, at least in part, excitatory input from HVC neurons projecting to Area X (HVCx neurons), which also exhibit sparse time-locked activity during song production (1–5 bursts/motif; Kozhevnikov and Fee, 2007; Goldberg et al., 2010; Fee and Goldberg, 2011; Woolley et al., 2014). In contrast to the precise temporal code of HVCx neurons, LMANx neurons exhibit more variable patterns of activity from song to song, despite a slight tendency to burst at particular points in song (Hessler and Doupe, 1999; Leonardo, 2004; Fee and Goldberg, 2011). While both HVCx and LMANx are thought to transmit efferent copies of premotor signals impinging on RA, HCVx is a likely source of the detailed timestamp for each moment in song in Area X, while LMANx is a likely source for information reflecting the variability of spectral features at each moment in song (Nixdorf-Bergweiler et al., 1995; Vates et al., 1997; Kozhevnikov and Fee, 2007; Prather et al., 2008; Fee and Goldberg, 2011).

Distinct from the temporal information transmitted from HVCx, we hypothesize that DLM conveys information about syllable onset/offset to Area X. DLM neurons appear to be strongly modulated at syllable onset and offset in young juvenile birds (<45 dph) when producing subsongs, with an average rate increase of 13.5 ± 1.9 Hz beginning 27.1 ± 6.2 ms prior to syllable onsets and average suppression of 8.2 ± 1.2 Hz beginning 40.0 ± 9.1 ms prior to syllable offset (Goldberg and Fee, 2012). This syllable-related activity persists after the subsong stage (> 45 dph) but may become less robust. Previously recorded DLM neurons are either identified LMAN-projecting neurons or suspected to be LMAN projecting. Future studies are needed to clarify whether DLM neurons shown to directly project to Area X carry syllable level representations. Of note, the origin of this putative syllable associated activity is thought to be driven by excitatory input from RA instead of inhibitory input from Area X (Goldberg and Fee, 2012), further strengthening the possibility that a thalamostriatal pathway might encode syllable-level representations that differ from corticostriatal inputs to Area X. Our suggestion that DLM neurons projecting to Area X carry syllable-level representations is consistent with the role of thalamostriatal pathways in initiation and terminating motor sequences. Lesions of DLM disrupt initiation of AFP-driven vocalizations (Chen et al., 2014). Further, the parafascicular (PFs) and the ventroposterior (VPs) neurons in the rat thalamus exhibit activity correlated with sequence initiation and execution, and corresponding thalamostriatal projections from these regions contribute to the smooth initiation and the appropriate execution of motor sequences (Díaz-Hernández et al., 2018).

### How Might the Thalamostriatal Pathway Contribute to Synaptic Plasticity in Area X and Guide Vocal Learning?

Although the neuronal basis for how Area X MSNs integrate their various inputs remains largely unknown, local credit assignment models provide at least one basis for thinking about how reinforcement learning shapes synaptic plasticity and guides song learning in Area X (Fee and Goldberg, 2011; Fee, 2012; Chen and Goldberg, 2020; Kornfeld et al., 2020). These models posit that coincident signals from HVC, LMAN, and VTA/SN, in a manner following three-factor Hebbian learning rules (Kuśmierz et al., 2017), drive plastic changes at corticostriatal synapses. MSNs are proposed to integrate information about timing from HVC, vocal variability from LMAN, and performance evaluation from VTA/SN to drive iterative changes in song performance through changes of synaptic weights at HVC corticostriatal synapses (Kornfeld et al., 2020). Coincident activation of HVC and LMAN inputs sets the learning window (eligibility trace) and occurring in the presence of elevated dopaminergic input leads to the strengthening of HVC synapses onto MSNs. These models provide a straightforward proposal for how moment-by-moment differences in performance might be linked *via* reward signals to assign credit to relevant corticostriatal synapses. However, in this framework, it is less clear how syllable-level information can be temporally assigned and chunked to permit learning and rearrangement of syllable sequences during song development.

**Figure 2:**
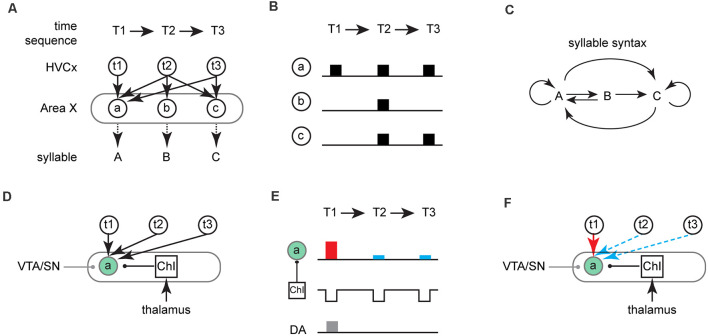
A gated-reinforcement learning model for sequence learning. **(A)** A schematic of the model for a three time-step (T1→T2→T3) song motif. Three ensembles of MSNs (a/b/c) in Area X (gray circle) are involved in learning the sequence of corresponding vocal elements (labeled as syllable A/B/C). MSN ensembles receive inputs from the ensemble of HVC neurons (HVCx, labeled as t1/t2/t3), each of which is active at the corresponding time-steps (T1–T3). Arrows between HVCx and an individual ensemble of MSNs indicate their synaptic connections and correspondent weights. In this hypothetical scenario, the probability of ensemble “a” of MSNs being activate is equal across all three time-steps for a given rendition of the song motif. This contrasts with ensemble “b” which can be activated exclusively at T2 or ensemble “c” which can be activated at either T2 or T3. **(B)** Theoretical neural activity in MSN ensembles (a/b/c) in Area X at different time steps given the corticostriatal connections shown in **(A)**. **(C)** Syllable syntax map showing the potential syllable transitions that could result from activity patterns across MSN ensembles in Area X shown in **(B)**. For instance, if ensemble “a” of MSNs is activate at all three time-step in one rendition, syntax “AAA” is produced. In another rendition, if ensemble “a” is activate at T1 and ensemble “c” is activated at T2 and T3, syntax “ACC” is produced. Arrows between different syllables indicate the corresponding transitions. Circle arrows besides “A” or “C” indicate repetition of the given syllable. **(D)** Schematic of the three inputs to the MSN ensemble “a”: VTA/SN, HVC, and ChIs. The square represents ChIs whose pauses are associated with syllable “A”. The green circle indicates that ensemble “a” is being depolarized at the same time that there are pauses in the activity of ChIs projecting onto ensemble “a”. Arrows indicate glutamatergic inputs from either HVC or DLM (thalamus). Lines with a circle at the end indicate modulatory inputs from either VTA/SN (gray, DA) or ChIs (black, Ach). **(E)** Schematic of gated-reinforcement learning. ChIs pauses are coincident with activation of ensemble “a” (green circle) while phasic dopamine signal (DA, gray rectangle) is active at T1. LTP (red rectangle) results at T1 when MSNs depolarization is coincident with ChIs pause and phasic increases in DA; LTD (blue rectangle) results at T2/T3 when MSNs depolarization is coincident with ChIs pause in the absence of phasic increases in DA. **(F)** Given the coincident activity of MSNs depolarization, cholinergic pauses and phasic increase in DA shown in **(E)**, LTP (red arrow) results at “t1→a” synapses and LTD (blue dashed arrow) results at “t2→a” and “t3→a” synapses.

We propose a gated-reinforcement learning model, which takes thalamostriatal input as well as ChI pauses into consideration to help resolve credit assignment for song syllables in vocal learning (Figures 1D, 2D). In this model, two independent components constitute the third factor used to modulate Hebbian plasticity in Area X: a reward prediction error signal, similar to that implemented in the above model, and a top-down feedback signal, often referred to as an attentional signal (Roelfsema and Van Ooyen, 2005; Rombouts et al., 2015; Kuśmierz et al., 2017). The first component is encoded by dopaminergic input from VTA/SN to Area X, reflecting positive and negative reward signals. This signal is delivered throughout Area X. The second component is a thalamostratial signal encoded by an efferent copy of signals from the motor cortical-like song circuit RA (Figure 1B). RA is topographically organized and neurons in the dorsal third of RA innervate premotor respiratory regions in the medulla, as well as DLM (Roberts et al., 2008; Goldberg and Fee, 2012). Therefore, the information conveyed *via* this pathway likely reflects expiratory and inspiratory timing information, which is tightly locked to syllable onsets (expiration) and syllable offsets (inspiration; Goller and Cooper, 2004). We propose that this putative input to Area X limits the occurrence of plasticity to affect only the corticostriatal synapses relevant to the actions selected within individual syllables and can be harnessed to help learn syllable transitions.

To walk through the model, imagine a simple song motif with three time-steps (T1-T3) and an ensemble of Area X projecting HVC neurons active during each of those time steps (HVCx, t1-t3). We hypothesize that Area X contains ensembles of MSNs which are responsible for the production of three distinct vocal elements that can ultimately be mapped onto the time-steps in the song motif during song learning (Figure 2A). For simplicity, we are only illustrating the corticostriatal input from HVC to Area X but envision an interaction between LMAN and HVC inputs onto MSNs as described in previous reinforcement learning models. Individual ensembles of MSNs receive inputs from the ensembles of HVC neurons and prior to sequence learning synaptic weights are equally distributed across all corticostriatal-MSN synapses (Figures 2A,B). Thus, a variety of syllable sequences can be produced across time-steps T1-T3 and which can lead to considerable variability in song syntax. For example, because all three ensembles of MSNs can be active at T2 and two ensembles of MSNs can be active at T3, six different potential syllable sequences can be generated in a motif (Figures 2B,C; T1-T3 = AAA, AAC, ABA, ABC, ACA, or ACC).

**Figure 3:**
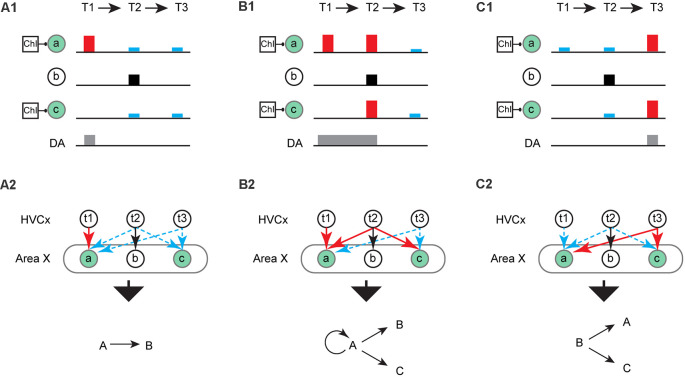
Hypothetical scenarios for learning syllable transitions. **(A)** A hypothetical scenario in which ChIs pauses are coincident with activation of ensembles “a” and “c” (green circle) and phasic increases in dopamine (DA, gray rectangle) occurs at T1. **(A1)** LTP (red rectangle) and LTD (blue rectangle) result at corticostriatal synapses on ensembles “a” and “c”, respectively. There is no change (black square) in the ensemble “b” when MSNs depolarization is not coincident with a pause in ChIs and/or phasic increases in DA. **(A2)** LTP (red arrow) results at “t1→a” synapses and LTD (blue dashed arrow) results at “t2/3→a” and “t2/3→c” synapses. Syllable “A” and “C” are omitted frequently at T2 and T3, resulting in reinforcement (learning) of the syllable sequence “A→B”. **(B)** Same as **(A)** but DA is active at T1 and T2 **(B1)**. **(B2)** Syllable “A” and “C” are omitted frequently at T3 due to the LTD at “t3→a/c” synapses. Consequently, the production of syllable sequences “A→A”, “A→C” or “A→B” is reinforced. **(C)** Same as **(A)** but DA is active at T3 **(C1)**. **(C2)** Syllable “A” is omitted frequently at T1 due to the LTD induced at “t1→a” synapses. Consequently, the production of syllable sequences “B→A” or “B→C” are reinforced.

We hypothesize that DLM provides strong input to ChIs (Figure 2D). Recession or decreases in excitation from the thalamus are associated with individual syllables and cause pauses in ChIs activity. These pauses create temporal windows for synaptic plasticity at corticostriatal-MSN synapses. Within these windows, the coincidence of cortical excitatory input and phasic dopamine regulates plasticity at corticostriatal synapses (Figures 2D,E). Long-term potentiation (LTP) can be induced at relevant synapses (t1→a) when a ChIs pause is coincident with MSNs depolarization (e.g., driven by HVC input) and phasic dopamine), while long-term depression (LTD) can be induced at synapses (t2/3→a) when a ChIs pause is coincident with MSNs depolarization alone (Figures 2E,F). MSNs depolarization with phasic dopamine and without ChIs pauses (e.g., synapse t2→b in Figures 3A–C), will result in no change in synaptic plasticity (Zhang et al., 2019). As illustrated in Figures 2D,E, the syllable “A” is reinforced at T1 but not at T2 or T3, because MSNs depolarization, cholinergic pauses, and DA activation are only synchronized at T1.

Building from this, we hypothesize that windows for plasticity regulated by ChIs might ultimately account for the ability to learn syllable sequences and the variety of sequence rearrangements typically observed during the song learning process. To illustrate this, consider when ChIs pauses are associated with ensembles “a” and “c” while a phasic increase in DA is restricted at T1. In this example, a syllable sequence “AB” can be reinforced as the dominant sequence, with syllable “C” or “A” largely omitted at T3 (Figure 3A). If a phasic increase in DA is extended to T2 while maintaining the same pattern of ChIs pauses, three different transitions may emerge (“AA” or “AC” and the less likely “AB”; Figure 3B). In another scenario, when phasic DA is restricted at T3, the syllable “A” is predicted to be omitted at T1 while syllable “C” or “A” may be produced at T3, resulting in two potential transitions (“BA” or “BC”; Figure 3C). Although increasingly speculative, a similar process might be used for inserting silent gaps or merging of song syllables through the removal of silent gaps. For example, we can imagine that vocal element “B” initially described in Figure 2A is a silent gap. This silent gap can be coincident with the activity of an ensemble of MSNs in Area X but will usually not be coincident with ChIs pauses (as depicted in the scenarios in Figure 3). If “B” is a silent gap, it is simple to see how a gap can develop before (Figure 3C2), or after a syllable (Figure 3A2). Similarly, two syllables “A” and “C”, for example, can be merged into a single syllable by removing the intervening gap between them.

Incorporating syllable-level ChI activity into existing basal ganglia reinforcement models addresses potential limitations of previous models and is supported by what is currently known about BG circuits in songbirds and mammals (Figure 1D). In mammals, ChIs may be faster to respond to changes in excitatory input than MSNs (Zhang et al., 2018). ChIs pause in response to receding excitatory input and resume tonic activity with subsequent excitatory input (Zhang et al., 2018). *In vivo* recording of putative ChIs in Area X of juvenile birds show that they exhibit activity peaks prior to syllable onsets and decreased activity during syllable production (Pidoux et al., 2015). The firing patterns of DLM neurons and ChIs, particularly the coincidence of rate changes relative to the syllable onset and offset (Goldberg and Fee, 2012; Pidoux et al., 2015), support our speculation that ChIs pauses are driven by transient decreases in excitatory input from DLM. In addition to providing a second factor controlling temporal windows for Hebbian plasticity, our model helps capture a little more of the known complexity of BG circuits, including cell types and known pathways. The pause response in striatal tonically active neurons, believed to represent ChIs, when coincident with phasic dopamine and depolarization of MSNs, could be sufficient for the induction of corticostriatal LTP *in vivo*, suggesting that ChIs pause might provide critical temporal constraints for the induction of plasticity in the BG (Zhang et al., 2019).

Several important questions remain regarding this proposed model. First, our model does not account for other known properties of thalamostriatal circuits. Aside from signaling through ChIs, thalamostriatal circuits also provide abundant direct inputs onto MSNs (Figure 1D; Lacey et al., 2007; Smith et al., 2009; Guo et al., 2015). Thus, thalamostriatal activity might have a significant direct influence on spike-timing-dependent plasticity (STDP) at corticostriatal synapses (Mendes et al., 2020). In addition, the distribution of cell types in Area X receiving direct input from DLM and/or DTZ remains to be examined. Second, our model undoubtedly oversimplifies how dopaminergic and cholinergic signaling can interact in the striatum. Although the interplay between dopaminergic and cholinergic neuromodulation in the striatum has been long established (Di Chiara et al., 1994; Threlfell et al., 2012; Straub et al., 2014; Zhang et al., 2018), the functional outcome of these interactions and their influence on MSNs at fast time scales needs further examination. Performing simultaneous manipulation and/or recording of both circuits in behaving birds will ultimately be needed to understand how these interactions relate to the proposed model. Lastly, we cannot exclude other possible mechanisms that can contribute to pauses in ChIs, such as midbrain dopamine input and/or GABAergic input from the midbrain or some other source (Lim et al., 2014; Zhang and Cragg, 2017; Ahmed et al., 2019).

## Discussion: Outlook and Future Directions

Understanding the role of thalamostriatal pathways in learning and production of vocal motor sequences has been limited by the lack of tools to map the functional organization of these circuits and methods to selectively monitor or modulate pathway-specific neuronal populations intermingled within BG circuits. This situation has changed dramatically with the advent of optogenetic and genetic lesioning approaches as well as progress in optical methods which can be used to monitor or manipulate selected subsets of neuronal populations embedded in the thalamus and striatum.

To help test the role of RA-DLM-Area X circuits in vocal learning and production, Cre (recombinase) dependent genetic lesioning experiments can be performed to selectively ablate DLM_X_ inputs in juvenile or adult birds. Similar approaches have been used to investigate the roles of intratelencephalic, corticostriatal, and dopaminergic pathways in vocal learning and production in zebra finches (Roberts et al., 2017; Hisey et al., 2018; Sánchez-Valpuesta et al., 2019) as well as the roles of the corticostriatal and thalamostriatal pathway during motor learning and execution in rats (Wolff et al., 2019). To test the role of ChIs in vocal learning and production, a Cre-dependent genetic lesioning strategy can also be used locally to eliminate ChIs in Area X. A novel vector has been developed to target transgene expression in ChIs in the monkey striatum (Martel et al., 2020). Either the vector can be used directly, or the choline acetyltransferase (ChAT) promoter can be assembled into an AAV construct to drive the expression of Cre in ChIs in Area X. Similar conditional expression of the transgene has been demonstrated to be efficient in Area X when the Cre-loxP system is delivered by two separate AAV constructs (Xiao et al., 2021). Alternatively, anti-ChAT conjugated saporin toxins, which are well established to specifically target and ablate ChIs in rodent striatum (Laplante et al., 2011; Aoki et al., 2015; Crevier-Sorbo et al., 2020), may provide a virus-free tool to eliminate ChIs in Area X.

To test the signals propagated in RA-DLM-Area X circuits, cell-type-specific calcium imaging approaches, which have previously been used to monitor the activity of HVC _RA_ neurons during courtship song production (Daliparthi et al., 2019), can be used to monitor the activity of DLM_X_ neurons during vocal learning in juvenile birds or vocal production in adult birds. Alternatively, axon-targeted GCaMP, which has been developed to enable *in vivo* imaging of thalamic boutons in deep cortical layers (Broussard et al., 2018), can be used to monitor the signal transmitted by DLM_X_ input in Area X.

Simultaneous monitoring of MSNs and ChIs has been achieved during movement in mice (Gritton et al., 2019). To monitor the interaction between MSNs and ChIs during vocal learning, a similar strategy can be adopted while replacing the ChAT-Cre line with the viral vector expressing pChAT-Cre (Martel et al., 2020). Lastly, axon targeted optogenetic excitation and perhaps inhibition can be used to test whether DLM provides strong excitatory input in ChIs and whether this input is sufficient to control the timing of pauses in ChI activity in Area X. As a complementary approach to genetic lesioning experiments, closed-loop optogenetic manipulation in behaving birds can be used to examine the real time contribution of DLM_X_ input in song learning and production with high temporal specificity. Similar approaches have been used to investigate the roles of the dopaminergic pathway in vocal learning and production in birds (Xiao et al., 2018, 2021).

Ultimately, detailed electrophysiological circuit dissection, employing optogenetic and cell-type-specific manipulations will be needed to gain a fuller perspective on circuit models for learning and controlling song syllables and song syllable sequences. With techniques currently in hand, these experiments are now feasible but will require a concerted effort across research groups to realize the full functional role of thalamostriatal circuits in vocal motor control and learning of motor syntax.

## Data Availability Statement

The original contributions presented in the study are included in the article, further inquiries can be directed to the corresponding author.

## Author Contributions

LX drafted and edited the manuscript. TR edited the manuscript. All authors contributed to the article and approved the submitted version.

## Conflict of Interest

The authors declare that the research was conducted in the absence of any commercial or financial relationships that could be construed as a potential conflict of interest.

## Publisher’s Note

All claims expressed in this article are solely those of the authors and do not necessarily represent those of their affiliated organizations, or those of the publisher, the editors and the reviewers. Any product that may be evaluated in this article, or claim that may be made by its manufacturer, is not guaranteed or endorsed by the publisher.
